# Cortical Blindness and Altered Mental Status following Routine Hemodialysis, a Case of Iatrogenic Cerebral Air Embolism

**DOI:** 10.1155/2018/9496818

**Published:** 2018-03-14

**Authors:** Lawrence Lau, Kory London

**Affiliations:** Thomas Jefferson University Hospital, 1020 Sansom Street, Room 1651, Thompson Building, Philadelphia, PA 19107, USA

## Abstract

Cerebral air embolism is a known complication from a myriad of iatrogenic causes. This case describes a 60-year-old female presenting from hemodialysis with altered mental status, bilateral homonymous hemianopia, and repetitive speech. A noncontrast head CT revealed air in the vein of Galen and the deep cerebral veins of the left thalamus and occipital sulcus, a complication from air being introduced into the patient via improper flushing of dialysis tubing. The retrograde flow of air bubbles in the upright patient during dialysis was likely responsible for the air embolus lodging in the cerebral vasculature. This patient was transferred to receive hyperbaric therapy, whereupon the patient survived with residual attention and spatial deficits.

## 1. Introduction

The introduction of air into the venous and arterial circulation is deadly or severely debilitating event that can occur during a variety of intravascular procedures, surgeries, and medical interventions across numerous medical and surgical specialties. The medical procedures that have the potential to introduce intravascular air are myriad and have been well described [1]. Life threatening issues such as decreased gas exchange, hypoxia, right ventricular strain, cardiac arrhythmia, and heart failure may present typically as pulmonary embolism and coronary occlusion; a focused history eliciting a high risk situation is imperatively raised the index of suspicion for an air embolus. Venous or arterial gas embolism may cause hyperemia and ischemia in end organ destinations which is devastating in coronary occlusion or in the cerebral vasculature [2]. Despite the advances in medical hardware, safety bundles, and procedural techniques, errors may still occur leading to devastating consequences. This case describes cerebral air embolism that occurred during a routine hemodialysis session through a patient's fistula.

## 2. Case Presentation

A 60-year-old female with a past medical history significant for chronic myeloid leukemia, end stage renal disease on hemodialysis, hypertension, chronic right foot osteomyelitis, and chronic pain syndrome/neuropathy presented for evaluation of altered mental status and acute visual loss. History was provided by Emergency Medical Services and the Dialysis Center and minimally from the patient secondary to altered mental status. The patient went to dialysis as per the patient's scheduled sessions, initially awake, alert, and oriented to person, place, and time. During dialysis, the patient reportedly became acutely “sleepy” and difficult to arouse. The patient when aroused complained of blindness in both eyes and exhibited repetitive speech. No other history was obtainable and no prior history of visual loss. The patient was disconnected from the dialysis unit and sent directly to the emergency department. Patient's vital signs in the emergency department were blood pressure 143/38 mmHg, heart rate 80 bpm, respirations 18/min, oxygen saturation 96% on room air, and afebrile (35.9°C). The exam was performed by the emergency medicine physician: patient exhibited normal ocular motions, sclera, and periorbital structures but exhibited bilateral homonymous hemianopia. No signs of acute trauma were noted. The patient was oriented only to herself and was moving all her extremities. Bilateral arms had AV fistulas with palpable thrills. The patient was given an NIH stroke scale of 8 (1 point for LOC questions, 1 point each for motor drift in each extremity, and 3 points for bilateral hemianopia). The rest of her exam was noncontributory.

**Figure 1:**
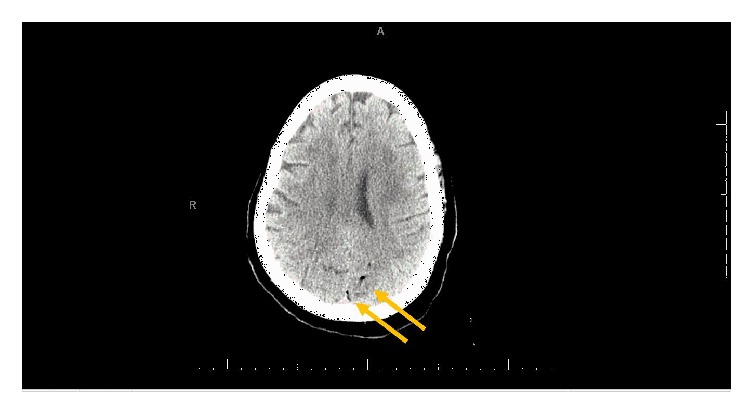
Multiple punctate and curvilinear foci of hypoattenuation in the left hemisphere, likely air within vascular structures given recent hemodialysis (gold arrows).

Given the nature of altered mental status and neurologic findings, an emergent noncontrast head CT was performed (Figure 1). CT was read as multiple punctate curvilinear foci of hypoattenuation regional to the vein of Galen and deep cerebral veins medial to the left thalamus and left occipital sulcus. Given recent hemodialysis, findings were consistent with air in vascular structures. The patient was placed on 100% oxygen via nonrebreather and placed in Trendelenburg position. Discussion with interventional neurosurgery was undertaken regarding potential for aspiration of air, but it was considered too diffuse and widespread to be efficacious. She was then transferred to a nearby hyperbaric center for definitive management. The patient was taken immediately by the hyperbaric team to the dive chamber, where she underwent a five hour session at 3 atm. MRI performed immediately after hyperbaric therapy demonstrated multiple subacute supra and infratentorial embolic infarctions in the bilateral occipital lobes, left thalamus, parietal lobe, and cerebellar tonsil, with minimal midline shift. Multiple areas showed sequela of small vessel and perfusional ischemic disease. The plan was to monitor for 4–6 hours followed by consideration of a second session if hemodynamic and neurologic assessment allowed/necessitated further care. The patient's inpatient stay was complicated by Klebsiella bacteremia and septic shock requiring intubation, multiple vasopressors, AV fistula malfunction, and runs of supraventricular tachycardia. No further hyperbaric therapy was performed for these reasons. The patient's mental status was further affected by hypoactive ICU delirium. A transthoracic echocardiogram with agitated saline did not demonstrate an intra-atrial communication. After a prolonged hospital course the patient's mental status improved. Evaluation by neurocritical care documented that patient was conversive, oriented to person and place, but was inattentive and needed constant redirection. The patient continued to have bilateral hemianopia. Extremities were full against gravity and sensation was intact. Of note, the patient was seen subsequently in the ED 3 months later for an unrelated visit by an author, her mental status had improved but she remained with severe visual deficits consistent with her bilateral homonymous hemianopia and had persistent difficulty with maintaining attention.

## 3. Discussion

The case described demonstrates an iatrogenic error where air was introduced into the patient's vasculature during dialysis. Dialysis related cerebral air embolism has been previously reported [3–11]. In this particular case, it was found that dialysate fluid was changed without halting the dialysis. A review of the literature demonstrates that gaseous bubbles can be introduced into the dialysis circuit in myriad ways; turbulent blood flow surrounding venous access sites, preexisting gas bubbles in dialysis tubing and dialyser, through introduction of air during connection/disconnection of dialysis tubing, and pressure or temperature gradients between patient and the dialyser which promote degassing of blood [12]. Though many safeguards and medical protocols exist on proper flushing of lines, catheters, and patient positioning, this complication remains possible and has proven to be deadly when emboli occlude pulmonary, cardiac, or neurologic vasculature [13]. In addition, hemodialysis devices, despite being fitted with air traps and ultrasonic detectors are not infallible in filtering microbubbles originating from luer lock connector tubing or from insufficient priming of dialysis hardware [12, 14, 15]. The bubbles may pass through the system without triggering the system alarm, especially when the bubbles are <50 *μ*L in diameter or flow rates are below the International Electrotechnical Commission standard for infusion pumps and dialysis machines, 0.1 ml/kg body weight for bolus infusion or 0.03 ml/kg/minute for continuous infusion [12, 16].

In a recent emergency department retrospective study, pneumocephalus from introduction of air into venous circulation appears in one in 3000 noncontrast head CTs [15]. Cerebral air embolus occurs when an air bubble travels through the venous system by rising up through the bloodstream in an upright or mobile patient. Air has a lower specific gravity than blood, which allows it to rise and lodge in cerebral vessels [17]. Air emboli can also travel into the arterial system via paradoxical embolization through a septal defect, pulmonary venous malformations, and a patent foramen ovale or from venous to arterial circulation if the pulmonary capillaries reach their maximum filtration rate and are unable to completely capture the air bubbles. A rare AV malformation may arise from a persisting fistulous connection of choroidal arteries when the median prosencephalic vein which does not regress normally could serve as another path for air emboli to enter the arterial circulation, though true incidence of vein of Galen malformation is reported at <1% [18]. Animal models have shown that the pulmonary capillary filtration system becomes overwhelmed when venous air emboli exceeds 0.35 ml/kg/minute, where bubbles are detected in 50% of animal arterial vessels by ultrasound at this flow rate [12]. These emboli lodged in the circulation will also trigger inflammatory and prothrombotic elements. In addition, air emboli irritate vascular walls, breaking down the glycocalyx endothelial surfaces and enhancing inflammation, platelet aggregation, and coagulation [12]. In a cerebral air embolus, microbubbles can irritate the vascular wall leading to breakdown of the blood brain barrier [2].

Cerebral gas embolism tends to occur in surgeries with cardiopulmonary bypass, upright craniotomy, hip replacements, and cesarean section, due to the presence of significant vascular beds that are incised during these operations [2]. In addition, the incised vessels have a hydrostatic gradient that favors intravascular entry of gas [1]. Additional case reports have noted pneumocephaly from injected air via peripheral IV placement [19]. Risk factors for developing cerebral air embolus during invasive vascular manipulations include hypovolemia, increased intrathoracic pressure in deep inspiration, and mobile or upright patients [2, 15, 20]. In this patient, the air embolus likely traveled retrogradely through the patient's vascular matrix into the cerebral vessels when the patient was seated upright during dialysis. Given that a follow-up echocardiogram showed no evidence of intra-atrial communication, a patent foramen ovale as the cause could be excluded.

The clinical symptoms of cerebral air embolus are myriad and can exhibit symptoms resembling stroke or acute cerebral infection. Fever, headache, abnormal ocular movements, vision loss, altered level of consciousness, hypotension, seizures, paralysis, and abnormal motor function have all been previously described [20–22]. A high index of suspicion must be held for air embolus in high risk patients, as the diagnosis relies mainly on the clinical picture. Studies on animal models, clinical case reports, and series of divers demonstrating neurologic/pulmonary sequelae have shown limited sensitivity of CT imaging in demonstrating the presence of cerebral air embolism. MRI, though able to detect subtle changes in brain edema, were also not shown to have the accuracy to rule out cerebral air embolism [2]. Therefore, a positive CT of the brain is not necessary to initiate treatment of cerebral air embolism if the clinical history is consistent [22].

Prospective long-term data has shown a 25% mortality rate in those affected from air embolus of any type, with 50% of survivors having permanent neurologic sequelae [22]. Treatment of cerebral air embolism follows similar guidelines for other air embolus syndromes: immediate discontinuation of hemodialysis procedures, 100% oxygen, and supportive care. Patients exhibiting seizures should be treated with benzodiazepines or barbiturates [17]. Air emboli may cause hemodynamic instability and should be addressed in patients with cardiopulmonary distress as well as cerebral symptoms. For other air embolism conditions, it has been suggested that positioning the patient in head-down Trendelenburg position would be beneficial in preventing intracardiac air from traveling out to the lungs by trapping it in the apex of the right ventricle; however, subsequent studies have demonstrated lack of clinical improvement with such maneuvers [20]. Patients with cerebral air embolism should especially avoid Trendelenburg positioning as this may potentially exacerbate cerebral edema [20]. In animal models, antithrombotic therapy with unfractionated heparin has shown improvement in neurologic impairment after experiencing cerebral air embolism [23]. Proposed definitive treatment of cerebral air embolus with neurologic findings involves hyperbaric oxygen therapy, as the high barometric pressure maintains oxygenation to ischemic tissues, denitrogenates cerebral tissue, and decreases cerebral edema mediated hypoperfusion. In addition, the high barometric pressure will dissolve air bubbles into the blood, removing their occlusive effect. Recent studies show that, for patients undergoing hyperbaric therapy for air embolus, beginning treatment with hyperbaric oxygen is most beneficial in the first six hours, reducing sequelae of tissue ischemia and death [22, 24].

Endovascular therapy is on the horizon as a potential treatment for cerebral air embolism. A single case report has documented the ability of using reperfusion techniques to access afflicted cerebral vessels, mechanically aspirating occluding air bubbles [25]. In addition, balloon assisted flow reversal, coupled with suction aspiration, has also been demonstrated [26]. The cases have resulted in improved or preserved neurologic function in afflicted patients. These techniques, though requiring additional study, may reveal an avenue towards invasive therapy to supplement our current supportive care approach.
